# The protease-sensitive N-terminal polybasic region of prion protein modulates its conversion to the pathogenic prion conformer

**DOI:** 10.1016/j.jbc.2021.101344

**Published:** 2021-10-25

**Authors:** Xiangyi Zhang, Yi-Hsuan Pan, Ying Chen, Chenhua Pan, Ji Ma, Chonggang Yuan, Guohua Yu, Jiyan Ma

**Affiliations:** 1 Key Laboratory of Brain Functional Genomics (Ministry of Education and Shanghai), Institute of Brain Functional Genomics, School of Life Sciences and the Collaborative Innovation Center for Brain Science, East China Normal University, Shanghai, China; 2 Fujian Provincial Key Laboratory for the Prevention and Control of Animal Infectious Diseases and Biotechnology, School of Life Sciences, Longyan University, Longyan, China; 3 Department of Neurodegeneraive Science, Van Andel Institute, Grand Rapids, Michigan, USA; 4 Chinese Institute for Brain Research, Beijing, China

**Keywords:** prion, N-terminal polybasic region, conformational change, POPG, convertibility, prion disease, DPI, days post inoculation, Fi, fluorescence intensity, NPR, N-terminal polybasic region of PrP, PK, proteinase K, POPG, 1-palmitoyl-2-oleoylphosphatidylglycerol, PrP, prion protein, recPrP, recombinant PrP, sPMCA, serial protein misfolding cyclic amplification

## Abstract

Conversion of normal prion protein (PrP^C^) to the pathogenic PrP^Sc^ conformer is central to prion diseases such as Creutzfeldt–Jakob disease and scrapie; however, the detailed mechanism of this conversion remains obscure. To investigate how the N-terminal polybasic region of PrP (NPR) influences the PrP^C^-to-PrP^Sc^ conversion, we analyzed two PrP mutants: ΔN6 (deletion of all six amino acids in NPR) and Met4-1 (replacement of four positively charged amino acids in NPR with methionine). We found that ΔN6 and Met4-1 differentially impacted the binding of recombinant PrP (recPrP) to the negatively charged phospholipid 1-palmitoyl-2-oleoylphosphatidylglycerol, a nonprotein cofactor that facilitates PrP conversion. Both mutant recPrPs were able to form recombinant prion (recPrP^Sc^) *in vitro*, but the convertibility was greatly reduced, with ΔN6 displaying the lowest convertibility. Prion infection assays in mammalian RK13 cells expressing WT or NPR-mutant PrPs confirmed these differences in convertibility, indicating that the NPR affects the conversion of both bacterially expressed recPrP and post-translationally modified PrP in eukaryotic cells. We also found that both WT and mutant recPrP^Sc^ conformers caused prion disease in WT mice with a 100% attack rate, but the incubation times and neuropathological changes caused by two recPrP^Sc^ mutants were significantly different from each other and from that of WT recPrP^Sc^. Together, our results support that the NPR greatly influences PrP^C^-to-PrP^Sc^ conversion, but it is not essential for the generation of PrP^Sc^. Moreover, the significant differences between ΔN6 and Met4-1 suggest that not only charge but also the identity of amino acids in NPR is important to PrP conversion.

Prion disease, also known as transmissible spongiform encephalopathy, is a large group of fatal neurodegenerative disorders affecting both humans and animals (1, 2). The normal prion protein (PrP^C^), which is essential for prion disease (3, 4, 5, 6), undergoes a conformational change to convert from the soluble and proteinase K (PK)-sensitive PrP^C^ to aggregated and PK-resistant PrP^Sc^ during the disease (7). Because of its seeding capability, the newly formed PrP^Sc^ is able to seed more PrP^C^-to-PrP^Sc^ conversion, which ultimately leads to neurodegeneration (8, 9, 10). Classic neuropathological changes in prion disease include spongiosis, gliosis, and deposition of aberrantly folded PrP (11).

Host-encoded PrP^C^ consists of a well-folded C-terminal domain with three α-helices and a short antiparallel β-sheet (12, 13) and an unstructured N-terminal polybasic region of PrP (NPR), an octapeptide repeat region, a central polybasic region, and a hydrophobic domain (Fig. 1*A*) (14). The pathogenic PrP^Sc^ conformer, however, consists solely of β-sheets (15, 16) and has a large PK-resistant C-terminal fragment (8, 17, 18). This PK-resistant fragment of PrP^Sc^ is sufficient to seed PrP^C^-to-PrP^Sc^ conversion and cause prion disease (19, 20).

**Figure 1:**
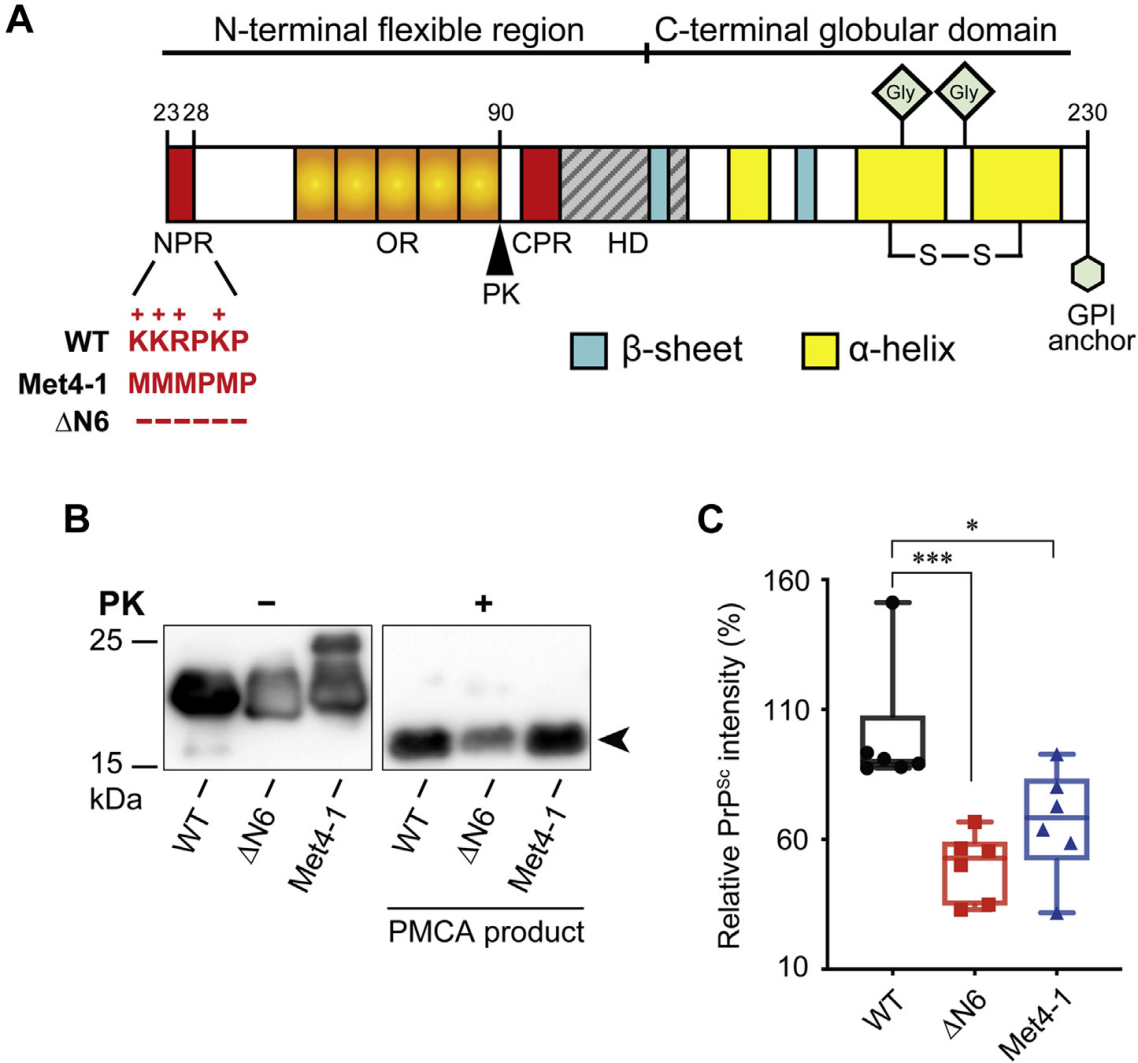
**Characteristics of WT and mutant recPrP.***A*, schematics of WT-recPrP, Met4-1-recPrP, and ΔN6-recPrP. *B*, representative images of one round of PMCA to generate recPrP^Sc^. PrP was detected by Western blotting with the 3F10 anti-PrP antibody. The signature PK-resistant band of recPrP^Sc^ was pointed by an *arrow*. *C*, densitometric analysis of PK-resistant bands in *B* (n = 6). The relative intensity of recPrP^Sc^ of WT sPMCA products (PK-resistant recPrP/recPrP without PK digestion) was set as 100%. Statistical significance was determined by one-way ANOVA followed by Tukey's multiple comparison test. ∗ represents *p* < 0.05; ∗∗∗ represents *p* < 0.01. Error bars indicate standard deviations. Single point in *C* was from a single PMCA reaction. PK, proteinase K; PMCA, protein misfolding cyclic amplification; recPrP, recombinant PrP.

Interestingly, several studies have shown that the NPR—which is located at very N terminus and remains PK sensitive in PrP^Sc^—plays a significant role in the pathogenesis of prion disease (21). Several transgenic mice expressing PrP with NPR deletion mutations (Δ23–31; Δ25–50; or Δ23–26) or replacement mutations (replacing ^23^KKRPK^28^ to KQHPH or AARPA) were established. While two deletion mutations (Δ25–50 and Δ23–26) did not significantly alter the disease process, other mutations (Δ23–31, KQHPH or AARPA replacement mutation) reduced the susceptibility to multiple prion strains (22, 23, 24). The alteration in susceptibility may result from the influence of NPR on PrP^C^-to-PrP^Sc^ conversion, the neurotoxic process, or both. The involvement of NPR in PrP-related neurotoxicity has been indicated by the several studies using organotypic brain slice and primary neuron cultures and the patch clamping studies of PrP-induced aberrant currents (25, 26, 27, 28).

Comparing to its role in neurotoxicity, the contribution of NPR to PrP^C^-to-PrP^Sc^ conversion is less clear. Because of its involvement in the endocytosis of PrP, it has been suggested that NPR may contribute to the recruitment of PrP^C^ to PrP^Sc^ (29). Using a serial protein misfolding cyclic amplification (sPMCA) system with CHO cell expressed PrP as the substrate, RML prion strain infected mouse brain homogenates as seed, and PrP-null mouse brain homogenates to provide the necessary cofactors, Miller *et al.* (30) showed that NPR deletion mutant is incapable of forming the PK-resistant PrP^Sc^ and suggested that NPR might be involved in binding to PrP^Sc^. Because the NPR-PrP^Sc^ binding assay was performed with crude brain homogenates (30), it cannot exclude the possibility that NPR may bind to other factors associated with PrP^Sc^. In addition, although the incapability of forming PrP^Sc^ in sPMCA is consistent with reduced or delayed PrP^Sc^ formation in prion-infected transgenic mice expressing various NPR mutants (22, 23, 24), it is incompatible with the fact that abundant PrP^Sc^ was formed in those mice. The use of a less robust sPMCA system likely accounts for the incompatibility.

Previously, we established an sPMCA system that robustly propagates recPrP^Sc^ with recPrP plus nonprotein cofactors—negatively charged phospholipid 1-palmitoyl-2-oleoylphosphatidylglycerol (POPG) and total RNA from mouse liver (31, 32, 33). The recPrP^Sc^ produced in this system recapitulates all the hallmarks of naturally occurring prions and causes prion disease in WT mice *via* various routes (34, 35). Using this sPMCA system, we studied the influence of NPR on PrP^C^-to-PrP^Sc^ conversion with two NPR mutants ΔN6 and Met4-1 (Fig. 1*A*). In ΔN6, all six amino acids in NPR were deleted. In Met4-1, four positively charged amino acids in NPR were replaced by structurally similar but neutral amino acid methionine. Our study revealed that both NPR mutants were able to form recPrP^Sc^ *via* sPMCA, but convertibility was reduced. Biochemical analysis of recPrP–POPG interaction showed that both mutants altered their interaction with POPG. Interestingly, clear differences in convertibility and POPG interaction were observed between ΔN6 and Met4-1, indicating that not only charges but also the identity of amino acids in NPR affects PrP conversion. Like WT recPrP^Sc^, the recPrP^Sc^ conformers formed by two NPR mutants caused prion disease in WT mice with a 100% attack rate. But the disease phenotypes were significantly different among mice inoculated with WT or either of the mutant recPrP^Sc^, indicating that there are conformational differences among these three types of recPrP^Sc^ aggregates.

## Results

### *In vitro* conversion of Met4-1 and ΔN6 recPrP to recPrP^Sc^

**Figure 2:**
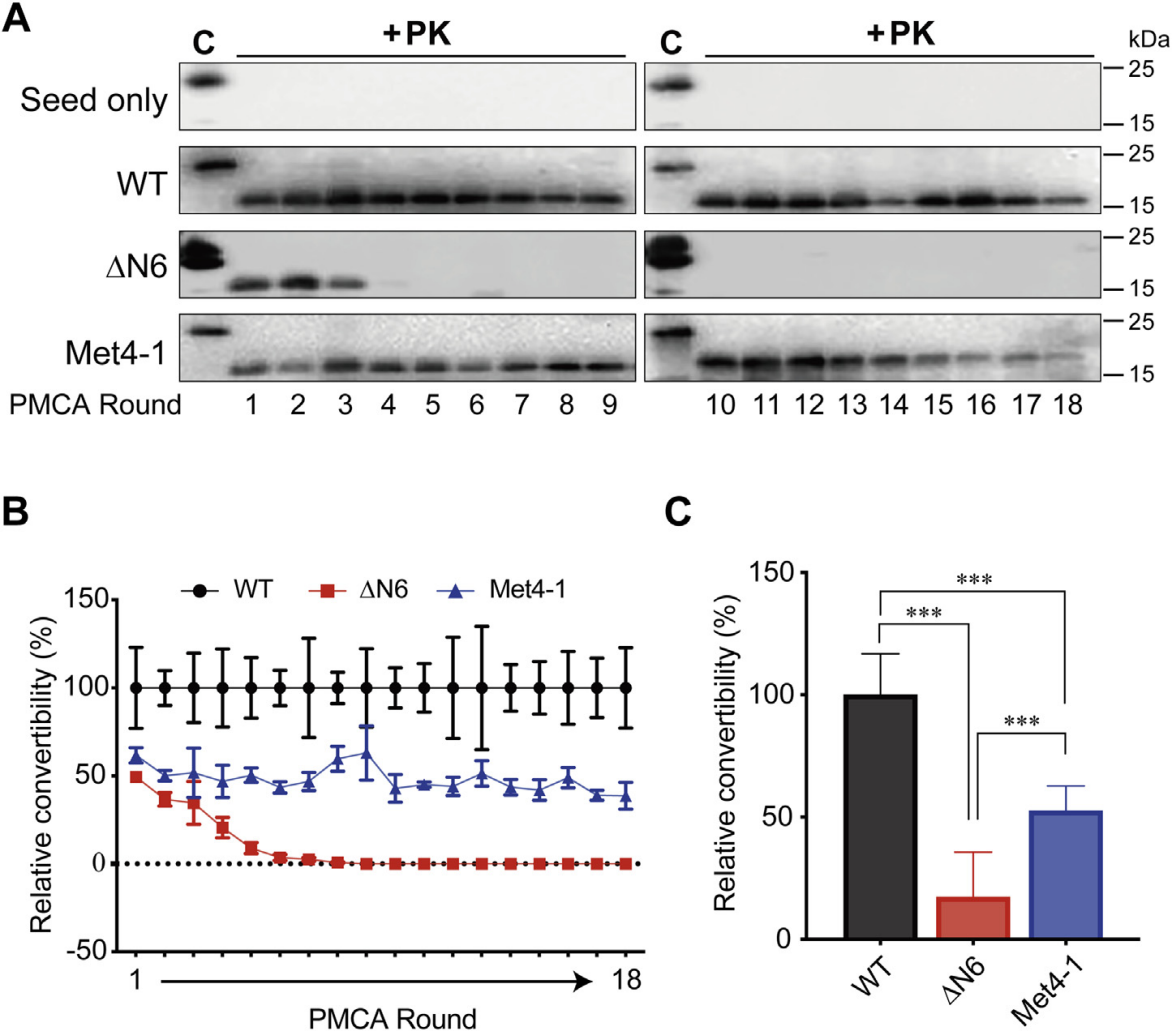
**Generation of recPrP**^**Sc**^**by sPMCA.***A*, representative images of 18 rounds of sPMCA (n = 4 independent sets of sPMCA propagations). Products of sPMCA were digested with PK and analyzed by Western blotting with the 3F10 anti-PrP antibody. The seed-only reaction was a negative control, in which recPrP in the substrate was replaced by double-distilled water (ddH_2_O). *B*, densitometric analysis of PK-resistant bands from *A* (n = 4). Relative convertibility = PK-resistant recPrP/recPrP without PK digestion. The relative convertibility of WT was set at 100%. *C*, mean convertibility of first nine rounds of sPMCA. The average convertibility WT was set as 100%. Statistical significance was determined by one-way ANOVA followed by Tukey's multiple comparison test. ∗∗∗ represents *p* < 0.01. Error bars indicate standard deviations. PK, proteinase K; recPrP, recombinant PrP; sPMCA, serial protein misfolding cyclic amplification.

To determine the influence of NPR on PrP conversion, we purified WT, ΔN6, and Met4-1 recPrPs and performed one round of PMCA seeded with previously generated WT recPrP^Sc^ (32). PK digestion of PMCA products revealed that all recPrPs were able to form PK-resistant recPrP^Sc^, but compared with WT, the ability of ΔN6 and Met4-1 to form recPrP^Sc^ was reduced (Fig. 1, *B* and *C*). To determine the influence of NPR mutations on serial recPrP^Sc^ propagation, 18 rounds of sPMCA were performed with 10 μl of previously generated WT recPrP^Sc^ to seed the first-round reaction. After each round, 10 μl of the product was used to seed next round PMCA and 30 μl of the products was subjected to PK digestion and Western blotting. In the control reactions, recPrP in the substrate was replaced with water, and no PK-resistant PrP was detected in the products of control sPMCA reactions (Fig. 2*A*, seed only), supporting that all PK-resistant PrP signals resulted from the conversion of recPrP in the substrate. Both WT and Met4-1 were able to propagate recPrP^Sc^ for 18 rounds of sPMCA, whereas ΔN6 formed recPrP^Sc^ only in the first five rounds and PK-resistant recPrP^Sc^ was barely detectable in fourth and fifth round products (Fig. 2*A*). Based on the intensity of PK-resistant band on Western blots, the recPrP^Sc^ produced by Met4-1 was approximately 60% of that of WT. ΔN6 was also able to convert to recPrP^Sc^ at ∼50% in the first round, but the conversion gradually reduced to 0% after the ninth round (Fig. 2*B*). When the average convertibility of first nine rounds of sPMCA were compared with that of WT (set as 100%), the relative convertibility of Met4-1 and ΔN6 to PrP^Sc^ was 50% and 20%, respectively (Fig. 2*C*).

Based on these results, we concluded that NPR significantly impacts the conversion from recPrP to recPrP^Sc^ in sPMCA, but recPrP with deletion or replacement NPR mutation are still able to form recPrP^Sc^. In addition, the significant difference between Met4-1 and ΔN6 suggested that not only the presence of positive charges but also the identity of amino acids in this region affects PrP conversion.

### Expression of WT, Met4-1, and ΔN6 PrPs in RK13 cells

The sPMCA provided a convenient *in vitro* system to study PrP conversion, but recPrP lacks the post-translational modifications and the conversion occurs in a test tube, which may not fully recapitulate the PrP conversion in a eukaryotic cell. To verify our findings, we established RK13 cell lines that stably expressed WT, ΔN6, or Met4-1 PrPs. RK13 is a rabbit kidney epithelial cell line that expresses little endogenous PrP and has been widely used to determine the susceptibility of various PrP forms to prion infection (36, 37).

**Figure 3:**
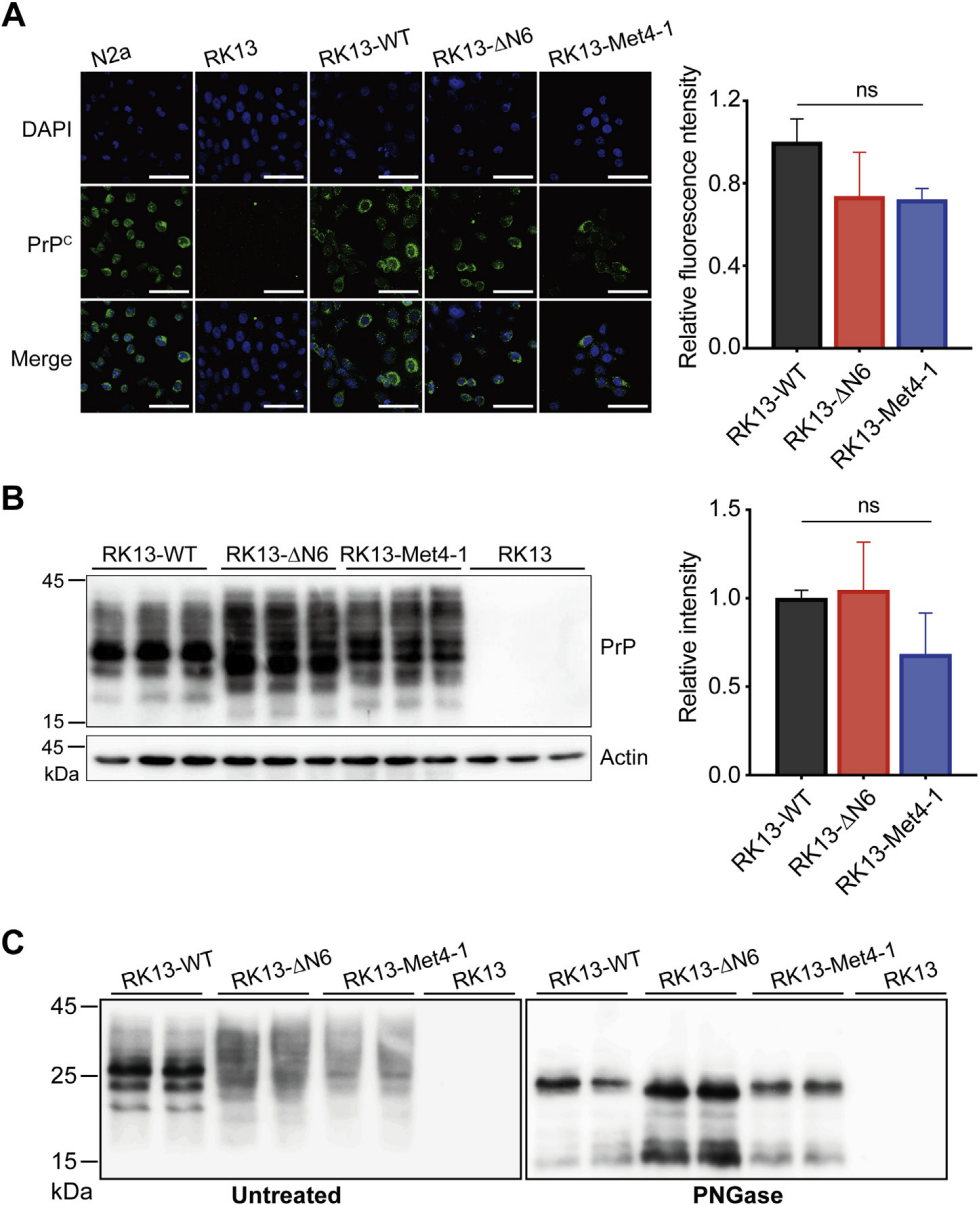
**Stable expression of WT-PrP, Met4-1-PrP, or ΔN6-PrP in RK13 cells.***A*, DAPI and immunofluorescence staining of stably transfected RK13 cells with the 3F10 anti-PrP antibody. N2a cells were used as the positive control, and untransfected RK13 cells were used as the negative control. Densitometric analysis of relative fluorescence intensity of 3F10 anti-PrP antibody-stained WT and mutant PrP proteins (n = 3, *right panel*). The relative fluorescence intensity of WT was set as 1.0. The scale bar represents 100 μm. *B*, Western blotting of RK13-WT, RK13-Met4-1, and RK13-ΔN6 cell lysates with 3F10 anti-PrP antibody (*left panel*) and densitometric analysis (*right panel*, n = 3). The average intensity of 3F10 antibody-stained bands of RK13-WT cells was set as 1.0. *C*, undigested (*left panel*) and PNGase-digested (*right panel*) RK13-WT, RK13-Met4-1, and RK13-ΔN6 cell lysates were examined by Western blots. Statistical significance was determined by one-way ANOVA followed by Tukey's multiple comparison test. ns represents no statistically significant difference. Error bars indicate standard deviations. DAPI, 4′,6-diamidino-2-phenylindole; PrP, prion protein.

Immunofluorescence staining was performed to compare the expression and localization of WT and mutant PrPs. While untransfected RK13 cells did not have any PrP signal, PrP staining was detected in cells transfected with WT, ΔN6, or Met4-1 (Fig. 3*A*). Since N2a neuroblastoma cell line is susceptible to prion infection (37), we used it as a positive control and found that the cellular localization of transfected PrPs was similar to that of endogenous PrP in N2a cells (Fig. S1). Densitometric analysis of immunofluorescence signals showed that RK13-Met4-1, RK13-ΔN6, and RK13-WT cells expressed a similar level of PrP (Fig. 3*A*), and this result was confirmed by immunoblot analysis (Fig. 3*B*). Treatment of cell lysates with peptide-N-glycosidase F converted the smearing PrP bands to a single major band of ∼25 kDa (Fig. 3*C*), confirming that similar to WT PrP, the NPR mutants were modified by N-linked glycosylation.

Collectively, our results revealed that the ΔN6 and Met4-1 mutations did not significantly alter the expression, cellular localization, and glycosylation of PrP. Importantly, the similar PrP expression level in RK13-Met4-1, RK13-ΔN6, and RK13-WT cells makes them suitable for comparing their susceptibility to prion infection.

### Prion infection in RK13-WT, RK13-Met4-1, and RK13-ΔN6 cells

**Figure 4:**
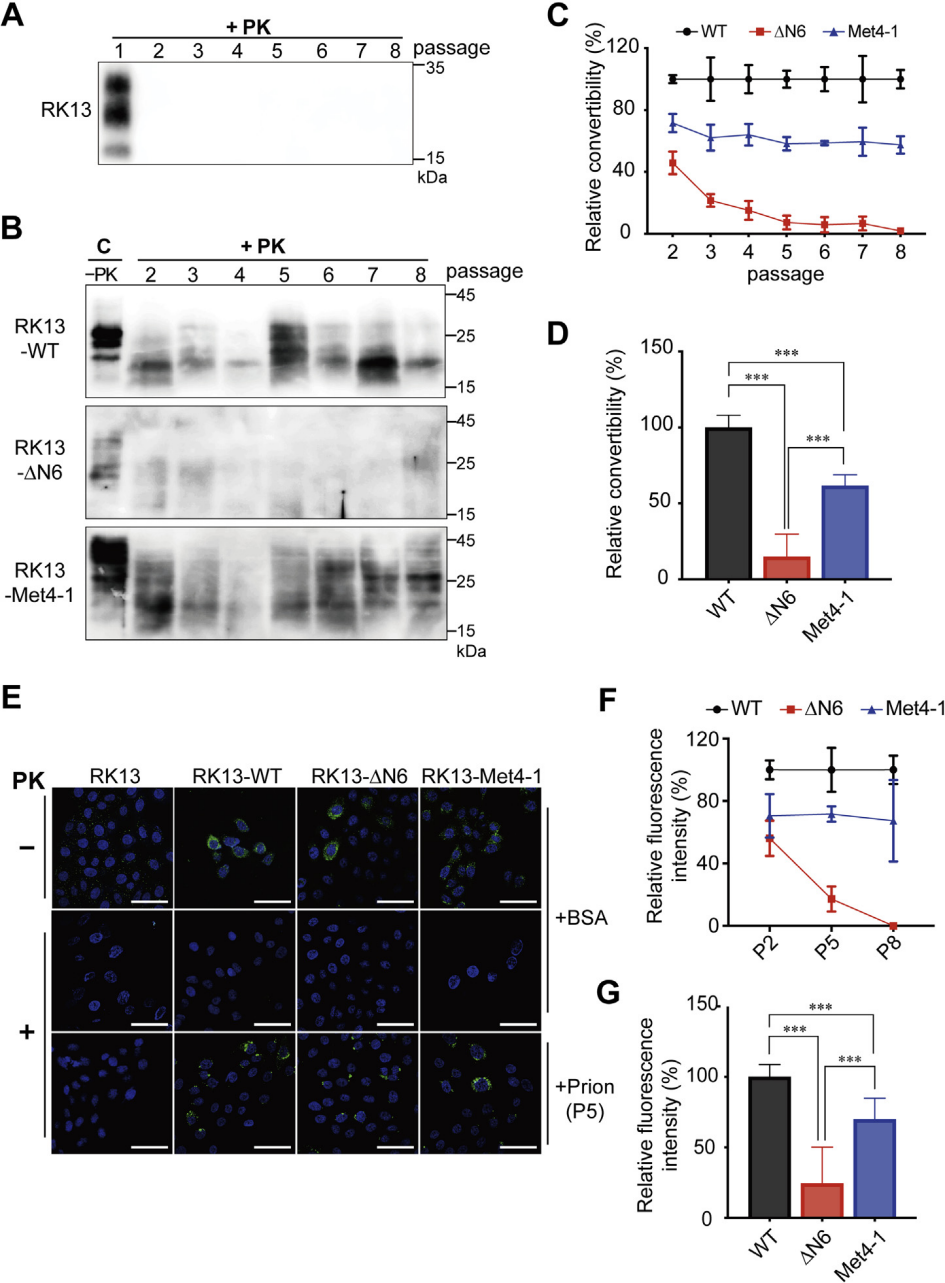
**Prion infection in RK13-WT, RK13-Met4-1, and RK13-ΔN6 cells.** RK13 cells expressing WT-PrP, Met4-1-PrP, or ΔN6-PrP were incubated with 0.5% brain homogenate prepared from a mouse suffering from terminal prion disease and then subjected to seven passages. The initial prion-infected culture was designated as passage 1 (P1). PK-resistant PrP^Sc^ in untransfected RK13 cells (*A*) and in WT-PrP, Met4-1-PrP, or ΔN6-PrP cells (*B*) were determined by PK digestion and Western blotting with the 3F10 anti-PrP antibody. *C* represents uninfected control cell lysates. Densitometric analysis of the PK-resistant PrP were shown in (*C*) and (*D*) (n = 3 independent prion infections of each stable RK13 cell line). Relative PrP convertibility = PK resistant PrP/PrP without PK digestion. The PrP conversion rate of RK13-WT cells was set as 100%. *E*, passage 5 (P5) cells were analyzed by immunofluorescence staining with the 3F10 anti-PrP antibody with and without PK digestion as indicated. Negative control cells were incubated with 0.5% BSA and analyzed in the same manner. The scale bar represents 100 μm. *F*, densitometric analysis of passages 2, 5, and 8 (P2, P5, and P8) cells that were immunofluorescently stained with the 3F10 anti-PrP antibody (n = 3 independent prion infections of each stable RK13 cell line). The relative fluorescence intensity of PK-resistant PrP in RK13-WT cells was set as 100%. *G*, comparison of mean relative fluorescence intensity of RK13-WT, RK13-Met4-1, and RK13-ΔN6 (n = 3). Statistical significance was determined by one-way ANOVA followed by Tukey's multiple comparison test. ∗∗∗ represents *p* < 0.01. Error bars indicate standard deviations. Results in *C* and *F* were from three independent prion infection experiments. PK, proteinase K; PrP, prion protein.

Stably transfected RK13 cells were infected with 0.5% brain homogenate prepared from a mouse succumbed to terminal prion disease. After that, cells were cultured for seven passages (P2–P8), and cell lysates of each passage were subjected to PK digestion and immunoblot analysis. Untransfected RK13 cells were subjected to the same treatment as the negative control, and in these cells, PK-resistant PrP^Sc^ was only detected in the first passage (P1) (Fig. 4*A*), confirming that the PrP^Sc^ in the inoculum was undetectable from passage 2. When stably transfected cell lysates were analyzed, the PK-resistant PrP^Sc^ was detected in RK13-WT or RK13-Met4-1 cells from all passages (P2–P8) but was barely detectable in RK13-ΔN6 cells (Figs. 4*B* and S2). Densitometric analysis revealed that compared with RK13-WT cells (set as 100% in each passage), the conversion rate (PK-resistant PrP/PrP without PK digestion) in RK13-Met4-1 cells was ∼60% in all passages, whereas the conversion rate in RK13-ΔN6 cells was decreased from ∼50% in P2 to 0% in P8 (Fig. 4*C*). The average PrP conversion rates in RK13-Met4-1 and RK13-ΔN6 cells were ∼62% and ∼15% of that of RK13-WT cells, respectively (Fig. 4*D*).

This finding was verified with immunofluorescence staining of P2, P5, and P8 cells (Figs. 4, *E*–*G* and S3). In this analysis, stably transfected cells incubated with 0.5% bovine serum albumin (BSA) were used as a negative control. As shown in Figure 4*E*, PK-resistant PrP signals were detected in RK13-WT and RK13-Met4-1 cells but were barely detectable in RK13-ΔN6 cells. Densitometric analysis showed that compared with RK13-WT cells (set as 100% in each passage), the PrP conversion in RK13-Met4-1 was ∼70% in all passages, whereas in RK13-ΔN6, the PrP conversion was ∼55% in P2, ∼20% in P5, and 0% in P8 (Fig. 4*F*). The average PrP conversion in RK13-Met4-1 and RK13-ΔN6 cells was ∼70% and ∼25% that of RK13-WT, respectively (*p* < 0.0001) (Fig. 4*G*).

Therefore, both prion infection assay in RK13 cells and the *in vitro* sPMCA assay support the impact of NPR on PrP conversion and the different convertibility between Met4-1 and ΔN6 PrP mutants.

### NPR mutations affect recPrP–POPG interaction

Negatively charged phospholipid POPG is a cofactor for generating recPrP^Sc^, and it binds recPrP *via* both electrostatic and hydrophobic interactions (38, 39). Since NPR is positively charged, we hypothesized that NPR mutations affected recPrP–POPG interaction and tested this possibility by comparing the binding of WT, Met4-1, and ΔN6 recPrPs to POPG using a discontinuous iodixanol gradient as previously described (38, 39, 40).

**Figure 5:**
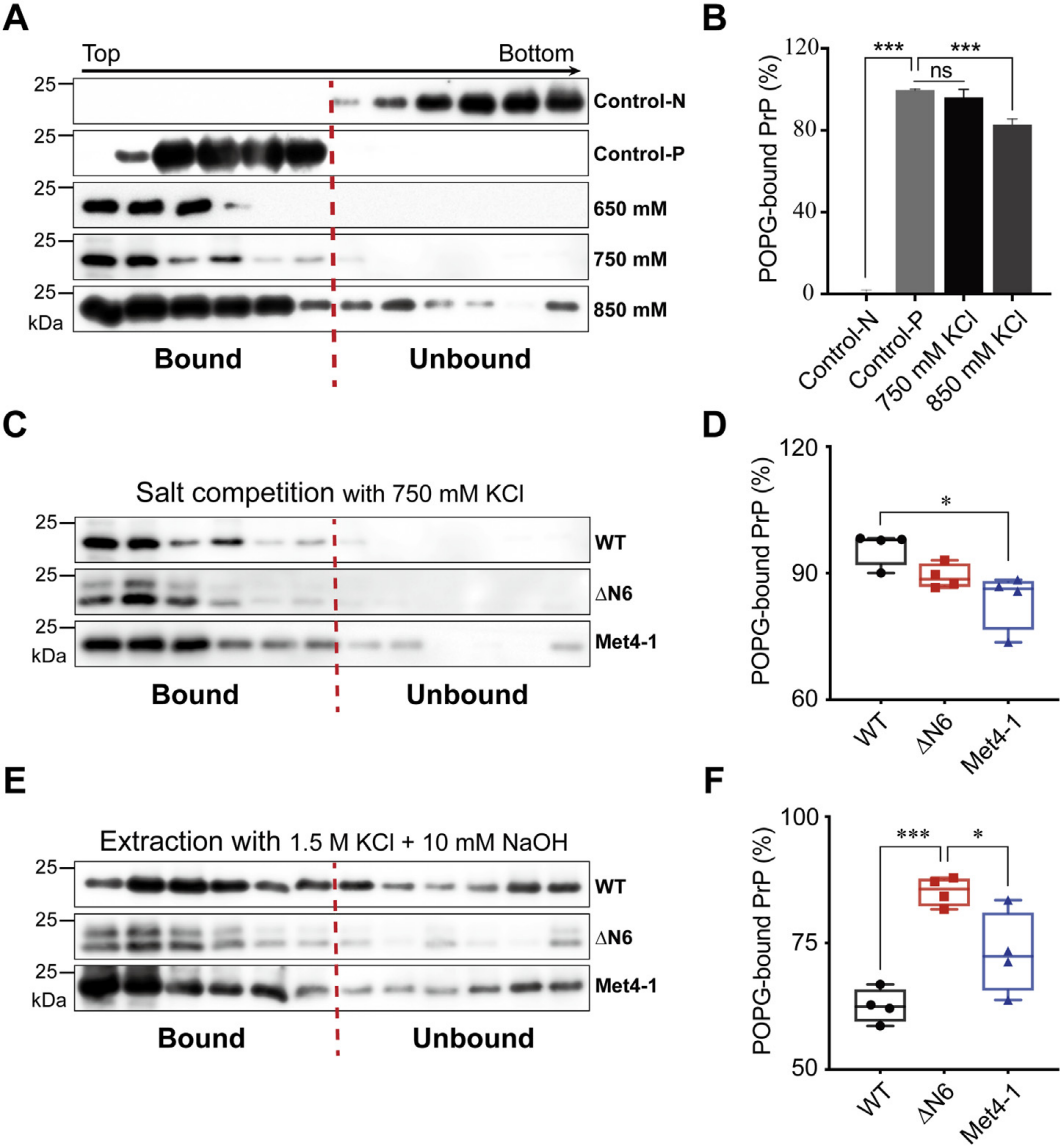
**The influence of NPR on recPrP–POPG interaction.***A*, salt competition analysis. WT-recPrP and POPG were separately incubated with 650, 750, or 850 mM KCl before being mixed and analyzed in the iodixanol density gradient centrifugation. The gradient was collected from top to bottom for a total of 12 fractions. Control-N: WT-recPrP without POPG incubation. Control-P: WT-recPrP with POPG incubation. *B*, densitometric analysis of the PrP bands in (*A*). The binding of control-P was set as 100%. *C*, to compare the POPG binding between WT and NPR mutants, 750 mM KCl competed binding of WT recPrP to POPG (fourth panel in *A*) was compared directly with that of Met4-1 and ΔN6 recPrPs, which went through the same procedure. *D*, densitometric analysis of Western blots as shown in *C* (n = 4). *E*, preformed WT-POPG, Met4-1-POPG, or ΔN6-POPG complex was incubated with 1.5 M KCl and 10 mM NaOH prior to the gradient analysis. *F*, densitometric analysis of Western blots as shown in *E* (n = 4). In all panels, PrP was detected by Western blotting with the 3F10 anti-PrP antibody. Statistical significance was determined by one-way ANOVA followed by Tukey's multiple comparison test. ∗ represents *p* < 0.05; ∗∗∗ represents *p* < 0.01; ns represents no statistically significant difference. Error bars indicate standard deviations. Every point in *D* and *F* was from an independent experiment. NPR, N-terminal polybasic region of PrP; POPG, 1-palmitoyl-2-oleoylphosphatidylglycerol; recPrP, recombinant PrP.

In this gradient, lipid-bound recPrP migrated to the top (Fig. 5*A*, control-P), whereas lipid-unbound recPrP remained at the bottom (Fig. 5*A*, control-N). Since recPrP–POPG interaction is at least partially because of the electrostatic interaction, the binding is influenced by high concentrations of salt (39). Indeed, the recPrP–POPG binding was not affected by the presence of 650 or 750 mM KCl, but when the KCl concentration increased to 850 mM, the binding was significantly reduced (*p* < 0.0001) (Fig. 5, *A* and *B*). The Met4-1 recPrP–POPG interaction, however, was significantly reduced in the presence of 750 mM KCl, resulting in ∼13% reduction compared with that of WT (Fig. 5, *C* and *D*). Interestingly, no reduction of ΔN6 recPrP–POPG interaction was detected at 750 mM KCl (Fig. 5, *C* and *D*).

To determine the influence of NPR on recPrP–POPG complex stability, recPrP–POPG complexes were treated with 1.5 M KCl and 10 mM NaOH (39). We found that the treatment dissociated Met4-1–POPG complex and the extent was similar to that of WT–POPG complex (Fig. 5, *E* and *F*). But the same treatment dissociated much less ΔN6–POPG complex and compared with WT recPrP, ∼40% more ΔN6 remained in the top fractions (Fig. 5, *E* and *F*).

One round PMCA with reduced POPG revealed that the conversion with WT recPrP was decreased (Fig. S4 and Table S1). Interestingly, PMCA with reduced POPG converted more NPR mutants to a PK-resistant form with a major PK-resistant band similar to that of recPrP^Sc^ (Fig. S4). Thus, reduced POPG in PMCA differently impacted the conversion of WT recPrP and NPR mutants. Together with the PrP–POPG binding results, this finding supported that NPR participates in PrP–POPG interaction and affects the POPG-facilitated recPrP-to-recPrP^Sc^ conversion.

### Pathogenicity of recPrP^Sc^ formed by Met4-1 and ΔN6

**Figure 6:**
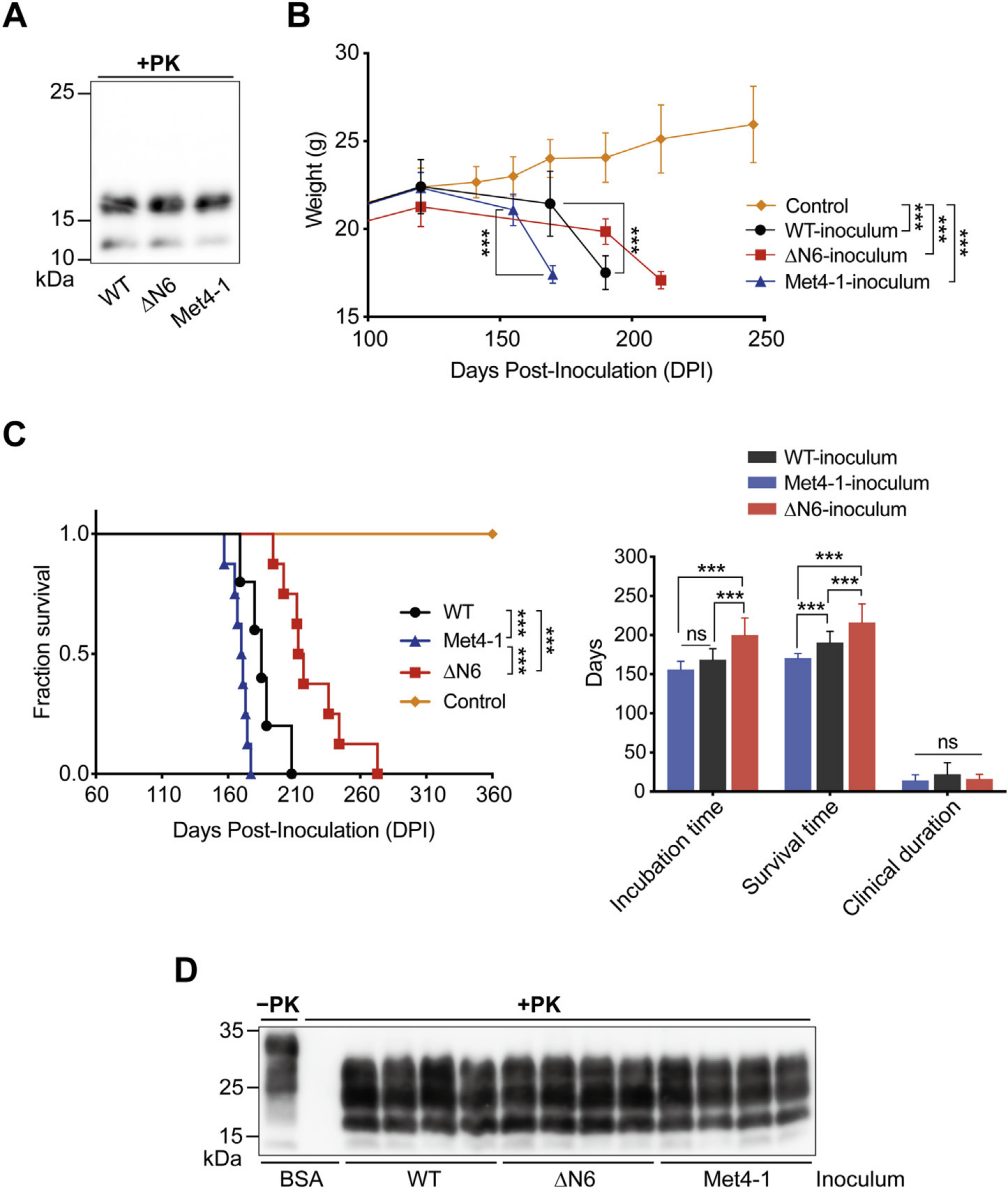
**Pathogenicity of WT, Met4-1, and ΔN6 recPrP**^**Sc**^**in WT mice.***A*, the PK-resistant PrP in the inoculum was detected by Western blotting with the 3F10 anti-PrP antibody. *B*, body weight changes in mice inoculated with indicated inocula. Control mice were inoculated with BSA in PBS (n = 4 mice). *C*, survival curve (*left panel*) and average incubation time, survival time, and the clinical duration as indicated (*right panel*) (n = 12 mice for each group). *D*, PK-resistant PrP^Sc^ in the brain cell lysates of mice inoculated with indicated inocula. PrP was detected by Western blotting with the 3F10 anti-PrP antibody. Statistical analysis for the survival curve in *C* was determined by log-rank test. The rest of the statistical analysis was determined by two-way ANOVA followed by Tukey's multiple comparison test. ∗∗∗ represents *p* < 0.01; ns represents no statistically significant difference. Error bars indicate standard deviations. BSA, bovine serum albumin; PK, proteinase K; recPrP, recombinant PrP.

To compare the pathogenicity of recPrP^Sc^ formed by WT and NPR mutants, the fourth round sPMCA products were collected. Based on the abundance of PK-resistant recPrP^Sc^ (Fig. 2*B*), adjusted amounts of fourth round sPMCA products were centrifuged, and pellets were resuspended in an equal volume of inoculum diluent. Immunoblot analysis confirmed a similar level of PK-resistant recPrP^Sc^ in all inocula (Fig. 6*A*).

WT mice intracerebrally inoculated with WT-recPrP^Sc^ lost ∼20% of body weight at ∼190 days post inoculation (DPI), and those intracerebrally inoculated with Met4-1-recPrP^Sc^ or ΔN6-recPrP^Sc^ lost ∼20% body weight at ∼170 and ∼220 DPI, respectively (Fig. 6*B*). The average incubation time of WT-recPrP^Sc^-inoculated mice was 168 ± 4.1 days, and the average survival time was 190 ± 4.1 days. The average incubation and survival times of Met4-1-recPrP^Sc^-inoculated mice were 156 ± 3.0 and 170 ± 1.7 days, respectively, and those of mice inoculated with ΔN6-recPrP^Sc^ were 199 ± 6.4 and 216 ± 6.9 days, respectively (Table 1). Although the incubation and survival times of mice inoculated with Met4-1-recPrP^Sc^ or ΔN6-recPrP^Sc^ were significantly different from that of WT-recPrP^Sc^-inoculated mice, the duration of clinical disease remained similar (Fig. 6*C*). As a control, WT mice were injected intracerebrally with 20 μl of inoculum diluent (1 mg/ml BSA in PBS) and monitored for 360 DPI. None of these control mice developed any clinical abnormality (Table 1 and Fig. 6*C*). As expected, PK-resistant PrP^Sc^ was detected in all diseased mice (Fig. 6*D*).

**Table 1:** Bioassay of recPrP^Sc^ in WT mice

Inoculum	Recipient mouse	Diseased mice/total mice	Incubation time^a^ (mean ± SEM, days)	Survival time^b^ (mean ± SEM, days)	Clinical duration^c^ (mean ± SEM, days)
BSA	C57BL/6j	0/4	—	>360	—
WT-recPrP^Sc^	C57BL/6j	12/12	168 ± 4.1	190 ± 4.1	22 ± 4.3
ΔN6-recPrP^Sc^	C57BL/6j	12/12	199 ± 6.4	216 ± 6.9	16 ± 1.7
Met4-1-recPrP^Sc^	C57BL/6j	12/12	156 ± 3.0	170 ± 1.7	14 ± 2.0

a Time from inoculation to the onset of diseases.

b Time from inoculation to the terminal stage of the disease.

c Time of clinical course.

**Figure 7:**
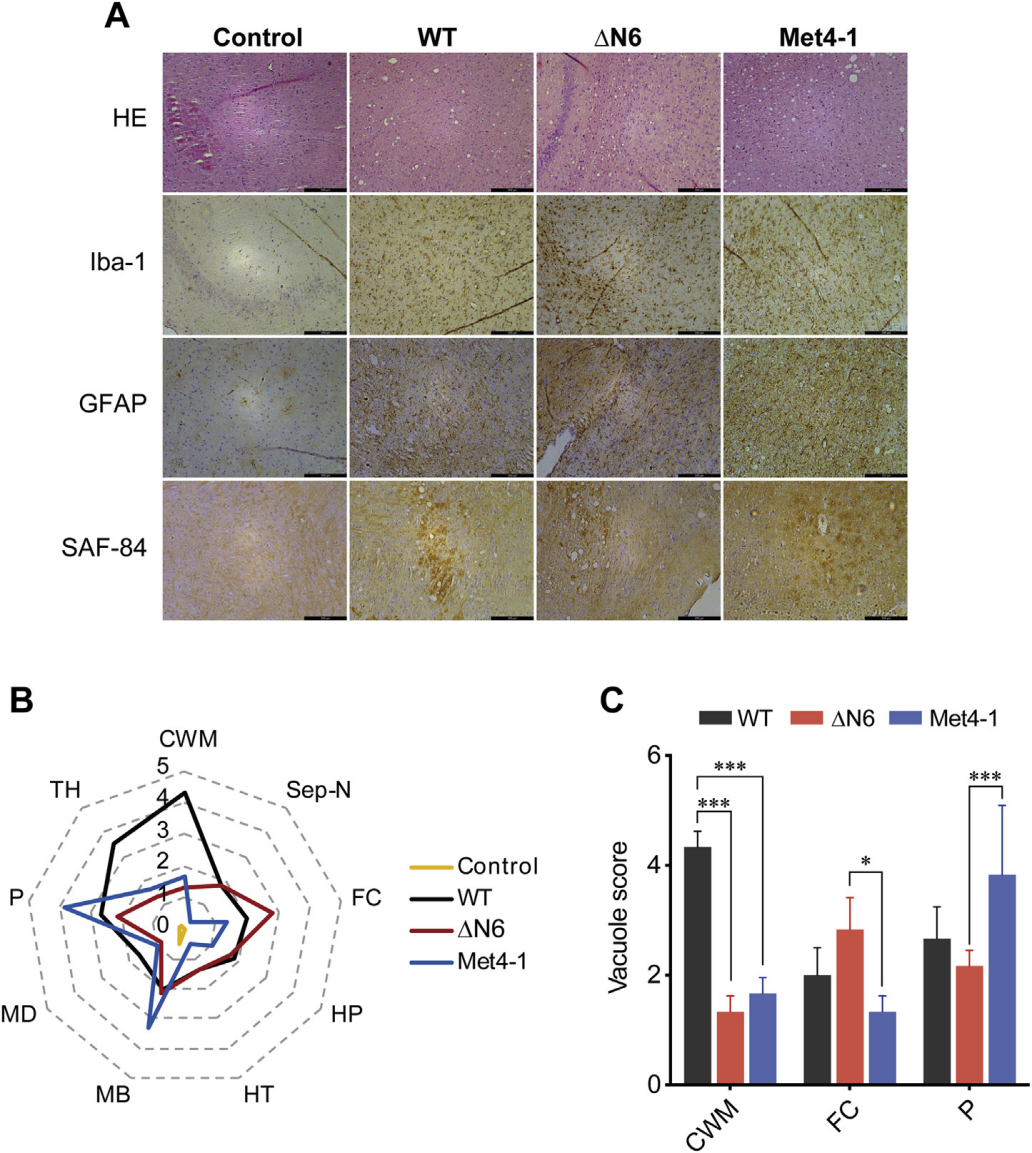
**Neuropathology of mice inoculated with WT, Met4-1, or ΔN6 recPrP**^**Sc**^**.***A*, brain sections were stained with HE, anti-Iba-1 antibody, anti-GFAP antibody, and SAF-84 anti-PrP antibody as indicated. The scale bar represents 200 μm. *B*, scores of vacuolization of various brain regions of mice inoculated with WT, Met4-1, and ΔN6 recPrP^Sc^. The following brain regions were examined: cerebellum white matter (CWM), septal nuclei (Sep-N), frontal cortex (FC), hippocampus (HP), hypothalamus (HT), midbrain (MB), medulla (MD), pons (P), and thalamus (TH). *C*, average vacuolation scores of CWM, FC, and P brain regions of mice inoculated with WT, Met4-1, or ΔN6 recPrP^Sc^ (n = 3). Statistical significance was determined by two-way ANOVA followed by Tukey's multiple comparison test. ∗ represents *p* < 0.05; ∗∗∗ represents *p* < 0.01. Error bars indicate standard deviation. recPrP, recombinant PrP.

Neuropathological analyses revealed classical spongiosis, astrogliosis, microgliosis, and the presence of aberrant PrP deposits in brains in all recPrP^Sc^-inoculated mice (Fig. 7*A*). Scores of spongiosis in various brain regions were significantly different among mice inoculated with WT, Met4-1, and ΔN6 recPrP^Sc^ (Fig. 7, *B* and *C*). The most severe spongiosis in WT-recPrP^Sc^-inoculated mice was in the cerebellum white matter, whereas the most severe spongiosis in Met4-1-recPrP^Sc^-inoculated and ΔN6-recPrP^Sc^-inoculated mice was in pons and frontal cortex, respectively (Fig. 7, *B* and *C*).

Collectively, our results revealed that recPrP^Sc^ formed by WT or NPR mutant recPrPs are fully infectious, causing prion disease in WT mice with a 100% attack rate, but with different disease phenotypes. Since prion disease phenotypes are determined by the structure of PrP^Sc^ (41), the differences among mice received WT, Met4-1, or ΔN6 recPrP^Sc^ suggest conformational differences among three types of recPrP^Sc^ aggregates.

## Discussion

High degree of evolutionary conservation and unique amino acid feature of NPR suggest that it plays an important role in the biological or pathological activity of PrP. Indeed, several studies revealed a significant influence of NPR on the pathogenesis of prion diseases (22, 24, 30). However, the mechanism of how NPR affects PrP^C^-to-PrP^Sc^ conversion remains unclear. Here, we showed that NPR significantly affects PrP conversion, but without NPR, recPrP is still capable of forming an infectious prion that causes prion disease in WT mice. A role of NPR in binding lipid cofactor for PrP conversion was supported by *in vitro* analyses of recPrP–lipid interaction, which plausibly explains its influence on PrP conversion. Interestingly, clear differences were detected between ΔN6 and Met4-1 in PrP conversion, lipid-binding capability, and pathogenicity of converted recPrP^Sc^ aggregates, which suggests to us that not only the positive charges but also the amino acid features of NPR are important for PrP conversion.

The role of NPR in PrP conversion was previously studied using sPMCA with CHO cell expressed PrP, and in that system, the NPR deletion mutant was unable to form PrP^Sc^ (30). Using sPMCA with bacterially expressed recPrP as the substrate, we found that recPrP with NPR mutations is capable of forming an infectious recPrP^Sc^, and this finding is consistent with the fact that PrP^Sc^ is abundantly formed in prion-infected transgenic mice expressing a variety of NPR mutants (22, 23, 24). The difference between these two studies could be due to the robustness of prion propagation in two sPMCA systems. In addition, we found that the ΔN6 deletion mutant has a greater reduction in convertibility compared with the replacement Met4-1 mutant. Because NPR deletion mutant was the only NPR mutant used in the previous study, the lower convertibility of this mutant may also contribute to the lack of PrP^Sc^ formation (30). Nevertheless, both studies support a great impact of NPR to PrP conversion.

A variety of reasons may account for the reduced convertibility of NPR mutants. Because cofactors are known to play an important role in PrP^C^-to-PrP^Sc^ conversion and most cofactors identified so far carrying negative charges, such as proteoglycan, nucleic acids, and negatively charged phospholipids (42, 43, 44), positively charged NPR likely contributes to the binding to these cofactors. We tested this hypothesis by analyzing the interaction between recPrP and negatively charged phospholipid POPG (38, 39). Surprisingly, the salt competition analysis—which presumably reflects the strength of electrostatic recPrP–POPG interaction—showed that Met4-1, but not ΔN6, mutation significantly reduced electrostatic interaction (Fig. 5, *C* and *D*). Since both mutations eliminate all the positive charged amino acids in NPR, this observation cannot be simply attributed to the reduction of positive charges. Given that the N terminus of PrP is known to interact with C-terminal region (28, 45, 46) and NPR deletion affects the folding and conformational stability of PrP (47), the ΔN6 deletion mutant may cause a subtle change in the C-terminal PrP conformation that compensates the removal of positive charges in NPR, resulting in the lack of reduction in electrostatic ΔN6–POPG interaction. Consistent with this theory, the conformational change in C-terminal regions to NPR may also alter the presentation of the hydrophobic domain, which is responsible for the hydrophobic recPrP–POPG interaction and a main contributor to the stability of the recPrP–POPG complex (48). Consequently, the stability of the ΔN6–POPG complex is enhanced. Further biophysical and structural studies are required to test these hypotheses.

The involvement of NPR in binding to negatively charged cofactors such as POPG also indicate that the previously reported reduced PrP^Sc^ binding by NPR deletion mutants (22, 30) may not be solely because of the direct NPR–PrP^Sc^ interaction. Instead, it could also be resulted from indirect binding of NPR to negatively charged cofactors that are associated with PrP^Sc^.

The shorter incubation time for Met4-1-recPrP^Sc^ caused disease in WT mice was unexpected. Since the PK-resistant PrP was normalized in all three inocula and disease duration was similar among three groups, this effect is unlikely because of the difference in the dosage of infectious recPrP^Sc^. A likely scenario could be that the unique Met4-1-recPrP^Sc^ conformation leads to a facile propagation in WT PrP in these mice, and/or the propagation of the Met4-1-recPrP^Sc^ conformation causes a severe neurotoxicity. Both could result in a shorter incubation time.

The obvious differences between Met4-1 and ΔN6 mutants in cofactor binding, convertibility, and pathogenicity are the most interesting findings of this study. It is possible that NPR mutations created an artificial prion transmission barrier, resulting in altered prion disease phenotypes in WT mice. But because the C-terminal PK-resistant portion of recPrP^Sc^ contains all the infectivity and is sufficient to cause prion disease in WT mice (20), we consider this to be an unlikely scenario. Because cofactors are known to influence the conformation of recPrP^Sc^ (44, 49), a more plausible explanation would be that the two mutations interact with cofactors differently, resulting in different recPrP conversion processes, leading to the generation of recPrP^Sc^ aggregates with different conformations that caused different disease phenotypes in mice. Notably, our conclusion that the identity of amino acids in NPR is important for PrP conversion is also consistent with a previous transgenic mouse study showing that replacement NPR mutant significantly altered the disease phenotypes (23).

Altogether, our study offers novel insights into the molecular mechanism of how NPR influences PrP^C^-to-PrP^Sc^ conversion and supports that the PK-sensitive N-terminus of PrP significantly influences the pathogenesis of prion disease.

## Experimental procedures

### Generation of recPrP mutants

The plasmid pET-22b-moPrPWT, which expresses the region encompassing amino acid residues 23 to 230 of murine PrP, was a generous gift from Dr Surachai Supattapone. This plasmid was used as the template to create PrP mutants Met4-1 and ΔN6.

To create Met4-1, a DNA fragment was generated by PCR using *Prnp* forward primer Met4-1-NdeI, 5′-GGGAATTCCATATGATGATGATGCCAATGCCTGGAGGGTGGAACACCGGTG-3′ and *Prnp* reverse primer R-XhoI/NotI, 5′-CGATACCGCTCGAGGCGGCCGCTCAGGATCTTCTCCCGTCGTAATAGG-3′. This primer set amplified the region encoding amino acid residues 23 to 230 of PrP with the sequence of the six amino acids in the NPR changed from KKRPKP to MMMPMP. The 624-bp PCR product was cloned into the vector pET-22b between NdeI and XhoI sites, generating the expression vector pET-22b-moMet4-1.

To create mutant ΔN6, a DNA fragment was generated by PCR using *Prnp* forward primer ΔN6-NdeI, 5′-GGGAATTCCATATGGGAGGGTGGAACACCGGTG-3′ and *Prnp* reverse primer R-XhoI/NotI, 5′-CGATACCGCTCGAGGCGGCCGC TCAGGATCTTCTCCCGTCGTAATAGG-3′. This primer set amplified the region encoding amino acid residues 29 to 230 of PrP with the six amino acids in the NPR deleted. The 606-bp PCR product was cloned into the vector pET-22b between NdeI and XhoI site, generating the expression vector pET-22b-moΔN6.

To produce WT, Met4-1, and ΔN6 recombinant PrP proteins, the vectors pET-22b-moPrPWT, pET-22b-moMet4-1, and pET-22b-moΔN6 were separately transformed into *Escherichia coli* BL21 (DE3) cells. The recPrPs were expressed and purified as previously described (31, 50). Purified recPrPs were subjected to centrifugation at 100,000*g* at 4 °C for 1 h to remove aggregated proteins, and then the protein concentration was determined using the BCA Protein Assay Kit (Thermo Fisher Scientific; catalog number: 23225).

### sPMCA

POPG (1-palmitoyl-2-oleoyl-*sn*-glycero-3-phospho-(1′-*sn*-glycerol) (sodium salt)) (Avanti Polar Lipids; catalog number: 840457) in chloroform was dried under a stream of nitrogen in a 42 °C water bath and then hydrated with 20 mM Tris–HCl buffer (pH 7.4) to a final concentration of 1.0 mg/ml. The hydrated POPG was repeatedly vortexed and sonicated until the solution became clear. The solution was then flushed with nitrogen and stored at 4 °C. Isolation of total liver RNA from FVB/NJ mice and preparation of sPMCA substrate with or without POPG were performed as described previously (32, 50, 51). For the seed-only negative control sPMCA reactions, recPrP in the substrate was replaced by double-distilled water. For the first round of sPMCA, 10 μl of previously generated WT-recPrP^Sc^ was used as the seed and mixed with 90 μl of substrate (WT, Met4-1, or ΔN6 recPrP) containing 16 μg of total liver mRNA and 2.3 μg of POPG. The mixture was subjected to 48 cycles of sonication for 30 s and incubation at 37 ^o^C for 29.5 min. For subsequent rounds of sPMCA, WT-recPrP^Sc^ seed was replaced with 10 μl of the product of the preceding sPMCA round.

### Detection of PK-resistant recPrP^Sc^ generated by PMCA

After each round of PMCA, 30 μl of the PMCA product was incubated with 10 μl of PK stock solution (100 μg/ml) for 30 min at 37 °C. The digestion was terminated by adding 5 mM PMSF and incubating on ice for 5 min. The proteins in each reaction were then precipitated by adding 20 μl of BSA (1 mg/ml) and 200 μl of ice-cold methanol and then incubating at −20 °C for 45 min. The mixtures were centrifuged at 20,000*g* for 30 min, and the pellet was resuspended in 15 μl of 1× SDS loading buffer (50 mM Tris–HCl, pH 6.8, 2% SDS, 2% β-mercaptoethanol, 10% glycerol, and trace amount of bromophenol blue). All samples were heated at 100 °C for 10 min and then cooled on ice for 1 min followed by a brief centrifugation at 7000*g* for 1 min. Samples were electrophoresed in 12% polyacrylamide Tris–HCl, SDS-PAGE gels, and then transblotted to Immobilon-P^SQ^ membranes (Merck Millipore; catalog number: ISEQ00010). In all experiments, recPrP without PK digestion was used as a control, which was 1/10th of the amount of recPrP used in each PK digestion. Each membrane was blocked with 5% nonfat milk in Tris-buffered saline buffer containing 0.1% Tween-20, reacted with 3F10 anti-PrP antibody that recognizes residues 137 to 151 of murine PrP (52, 53), and then incubated with antimouse IgG horseradish peroxidase–labeled goat antibody (Bio-Rad; catalog number: 1706516). All blots were reacted with ECL-plus reagent (Wanlei bio; catalog number: WLA006b) and analyzed by ImageQuant LAS400 (GE HealthCare). The relative intensity of the recPrP^Sc^ band was calculated by the formula: relative PrP^Sc^ intensity (Ri) = (+PK_intensity_)/(−PK_intensity_). In the experiments described in Figure 5, *G* and *H*, the relative PrP^Sc^ intensity in PMCA with reduced amount of POPG was calculated by the formula: (Ri without POPG − Ri with POPG)/Ri with POPG.

### Generation of stably transfected RK13 cell lines

RK13 rabbit epithelial cells (American Type Culture Collection CCL-37) were transfected with mammalian expression vector pcDNA3.1(+)-moPrPWT to express the full-length PrP^C^. A separate set of RK13 cells was transfected with pcDNA3.1(+)-moMet4-1 or pcDNA3.1(+)-moΔN6 to express mutant PrP Met4-1 and ΔN6, respectively. These expression vectors were constructed by Generay Biotechnology. All plasmids were linearized by digestion with ScaI (Thermo Fisher Scientific; catalog number: FD0434) before transfection. All transfected cells were selected with 600 to 1200 μg/ml of Geneticin (Gibco; catalog number: 11811023) in Dulbecco's modified Eagle's medium (Gibco; catalog number: 11965092) supplemented with 10% (v/v) heat-inactivated fetal bovine serum (Bovogen; catalog number: SFBS), 1% (v/v) l-glutamine (BBI Life Sciences), and 1% (v/v) penicillin/streptomycin (BBI Life Sciences; catalog number: E607011) as described previously (54).

### Prion infection of RK13 cells

Prion infection of transfected RK13 cells were performed according to previously reported protocols (54, 55) with minor modifications. Cells were plated in a 6-well plate to reach 50 to 70% confluence. After washing with 1× PBS, cells were incubated with 1 ml/well of 0.5% of mouse prion brain homogenate (prepared from a mouse suffering from terminal prion disease) in a 37 °C CO_2_ cell culture incubator for 5 h. The prion brain homogenate was preheated at 80 °C for 30 min to inactive proteins other than PrP^Sc^. Complete Opti-MEM was then added to each well, and the cells were continually cultured for seven passages with a 1:10 split for each passage. The original prion-infected culture was considered as passage 1. To prepare cell lysate, cells in 6-well plates were lysed in cell lysis buffer containing 10 mM Tris–HCl, pH 7.6, 100 mM NaCl, 10 mM EDTA, 0.5% NP-40 (v/v), 0.5% sodium deoxycholate (w/v), and 1 mM PMSF.

For immunofluorescence staining, cells of passages 2, 5, and 8 were seeded in wells of a 24-well plate and stained with 3F10 anti-PrP antibody. The control cells were incubated with 1 ml of 0.5% BSA in a manner identical to prion infection.

### Peptide N-glycosidase F treatment

Cell lysates were subjected to five cycles of freezing–thawing (in liquid nitrogen and at 37 °C) and then incubated with 0.025 U Benzonase (Merck Millipore; catalog number: 101654) in the presence of 20 mM MgCl_2_ at 37 °C for 30 min to degrade nucleic acids. The protein concentration in each cell lysate was then adjusted to 20 mg/ml. An aliquot of a cell lysate containing 25 μg of protein was incubated with peptide N-glycosidase F (New England Biolabs; catalog number: P0704S) in G7 reaction buffer (New England Biolabs; catalog number: B3704S) containing 1% NP-40 at 37 °C for 1.5 h. The deglycosylated samples were then subjected to SDS-PAGE and Western blotting with 3F10 anti-PrP antibody.

### PK digestion of cell lysates

To detect PK-resistant PrP^Sc^, 5 μl of cell lysates containing 100 μg of protein was incubated with 3 μl of PK stock solution (150 μg/ml) and 7 μl double-distilled water to reach a final PK concentration of 30 μg/ml. The digestion was carried out for 1 h at 37 °C followed by adding PMSF to 5 mM and incubating on ice for 5 min to terminate the reaction. PK-digested cell lysates were then analyzed by SDS-PAGE and Western blotting as described previously. The relative PrP convertibility was calculated based on the intensity of the PrP bands with and without PK digestion on Western blot and by the formula: relative convertibility = (+PK_intensity_)/(−PK_intensity_).

### Immunofluorescence staining of prion-infected RK13 cells

Immunofluorescence staining of prion-infected cells was adapted from previously reported protocols (56, 57) with some modifications. Cells were grown on poly-d-lysine-coated coverslips in a 24-well plate. At approximately 50% confluence, cells on coverslips were gently washed twice with 1× PBS, fixed in 4% paraformaldehyde at room temperature for 30 min, and then permeabilized with 0.5% Triton X-100 for 5 min. To detect PrP^Sc^, cells were digested with 20 μg/ml of PK at 37 °C for 10 min and incubated with 2 mM of PMSF for 15 min to terminate the reaction, and then incubated with 6 M guanidine hydrochloride at room temperature for 15 min. After that, cells were blocked with 5% normal goat serum in 1× PBS and then reacted sequentially with 3F10 anti-PrP antibody and then goat antimouse IgG Alexa Fluo-488 (Invitrogen; catalog number: A10680). Nuclei were stained with 1 μg/ml 4′,6-diamidino-2-phenylindole (Invitrogen; catalog number: D3571). Confocal laser scanning microscopy (Fluoview FV10i; Olympus) was performed. The fluorescence intensity (Fi) of stained PrP proteins was calculated as total Fi/cell numbers using ImageJ (National Institutes of Health). Relative PrP convertibility was calculated as Fi(+PK)/Fi(−PK).

### Animal ethics statement

Animal experiments were carried out in accordance with the Guidelines for the Care and Use of Laboratory Animals of China Ministry of Science and Technology. All procedures were approved by the Institutional Animal Welfare and Care Committees of Wuhan University and East China Normal University. All experiments were performed in authorized biosafety level-2 laboratories at East China Normal University (assurance number: m20191204).

### Preparation of recPrP^Sc^ inoculum and intracerebral inoculation

To purify WT recPrP^Sc^ inoculum, 500 μl of pooled fourth round sPMCA products was laid on 100 μl 10% sucrose cushion (w/v, in sterile 1× PBS) and centrifuged at 150,000*g* at 4 °C for 1 h. The resulting pellet was resuspended in 500 μl sterile 1× PBS and subjected to another round of ultracentrifugation on sucrose cushion. The final pellet was resuspended in 500 μl of inoculum diluent (1% BSA, w/v, in sterile 1× PBS) and stored at −80 °C. To prepare Met4-1 and ΔN6 inocula, ∼1000 and ∼2500 μl of pooled fourth round sPMCA products, respectively, were subjected to the same treatment described previously for WT recPrP^Sc^ inoculum. After the final centrifugation, the pellet of each mutant recPrP^Sc^ was resuspended in 500 μl of inoculum diluent.

For intracerebral inoculation, the inoculum was thawed and sonicated in an ice water bath for 5 min. Each 5- to 6-week-old female C57BL/6j mouse (Shanghai SLAC Laboratory Animal Co, Ltd) was intracerebrally inoculated with 20 μl of the inoculum as previously described (50). The inoculated mice were monitored daily for signs of prion disease and considered sick if three or more of the following symptoms were observed: emaciation, kyphosis, stiff tail, leg paresis, decreased activity, ataxic gait, clasping, head twitching, ruffled body hair, uracratia, lethargic, and cachexia. Mice were sacrificed once body weight loss reached ∼20%.

### PK digestion of mouse brain homogenates

The brain of each sacrificed mouse was dissected sagittally. Half of the brain was homogenized in sterile 1× PBS to prepare 10% brain homogenate (w/v). Triton X-100 and sodium deoxycholate were added to a final concentration of 0.5% each. Protein concentration in each brain lysate was determined using the BCA Protein Assay Kit (Thermo Fisher Scientific; catalog number: 23225). To detect PrP^Sc^, an aliquot of a brain lysate containing 50 μg of protein was digested with 30 μg/ml of PK for 1 h at 37 °C and then subjected to SDS-PAGE and Western blotting with 3F10 anti-PrP antibody.

### Histopathology and immunohistochemistry

Three mice from each group that had survival times closest to the average survival time of the group were chosen for the histopathological analyses. Brain sections of 5 μm were stained with hematoxylin and eosin Y (CoWin Biosciences) for histopathological examinations, anti-IbaI antibody (Wako; catalog number: 019-19741) to detect microglia, anti-GFAP antibody (Cell Signaling Technology; catalog number: 3670) to detect astroglia, and anti-SAF-84 antibody (Cayman; catalog number: 189775) for aberrant PrP deposit. For each mouse, at least one picture of 10× magnification was taken for each brain region shown in Figure 7*B*. For each picture, four random 200 × 200 μm squares were chosen to count the number of vacuoles. The semiquantitative score of spongiosis (33, 58) was based on the criteria listed in Table S2.

### Iodixanol gradient analysis

The discontinuous iodixanol density gradient was prepared with OptiPrep (Axis-Shield; catalog number: 1114542). Purified WT-recPrP, Met4-1-recPrP, and ΔN6-recPrP were separately mixed with POPG (1 mg/ml) at a molar ratio of recPrP:POPG = 1:1000. Each mixture was incubated at room temperature for 20 min and then mixed with OptiPrep to generate 500 μl of 36% iodixanol solution and placed in the bottom of a 2-ml ultracentrifuge tube (Beckman). To prepare the gradient, 500 μl of 31% and 200 μl of 5% iodixanol solution were sequentially layered on top of the 36% iodixanol solution. The gradient was centrifuged at 200,000*g* at 4 °C for 3 h and then collected from top to bottom for a total of 12 fractions of 100 μl each. Proteins in each fraction were precipitated by adding four volumes of ice-cold methanol and incubating at −20 °C for 45 min. The precipitate was collected by centrifugation at 20,000*g* for 30 min and then subjected to SDS-PAGE and Western blotting with 3F10 anti-PrP antibody (38, 39, 40). The binding capability of PrP^C^ to POPG was calculated as: total density of fractions 1 to 6 (F1–F6)/total density of fractions 1 to 12 (F1–F12). For salt competition, recPrP and POPG were separately incubated with KCl at indicated concentration for 10 min at room temperature, then mixed together, and incubated at room temperature for 20 min. The final mixture was subjected to the discontinuous iodixanol gradient assay. For the extraction assay, preformed recPrP^C^–POPG complexes were incubated with 1.5 M KCl and 10 mM NaOH at room temperature for 20 min and then subjected to the gradient analysis.

### Statistical analysis

Data were presented as means plus/minus standard deviations except for those were specifically indicated. The means were compared by ANOVA followed by Tukey's multiple comparison test. The survival curves of infected mice were analyzed using the log-rank test. Statistical analyses were performed with GraphPad Prism 7.0 (GraphPad Software, Inc). A *p* value <0.05 was considered statistically significant and was marked with a single asterisk (∗), and a *p* value <0.01 was marked with triple asterisks (∗∗∗).

## Data availability

All data are contained within the article and accompanying supporting information.

## Supporting information

This article contains supporting information (38, 39).

## Conflict of interest

The authors declare that they have no conflicts of interest with the contents of this article.
