# Supplementary material for: The protease-sensitive N-terminal polybasic region of prion protein modulates its conversion to the pathogenic prion conformer
Source: J Biol Chem. 2021 Oct 25;297(5):101344. doi: 10.1016/j.jbc.2021.101344 (PMC8604679; doi:10.1016/j.jbc.2021.101344)
Supplement: Supporting information [file mmc1.pdf]

SUPPORTING INFORMATION

Supporting figures

Figure S1

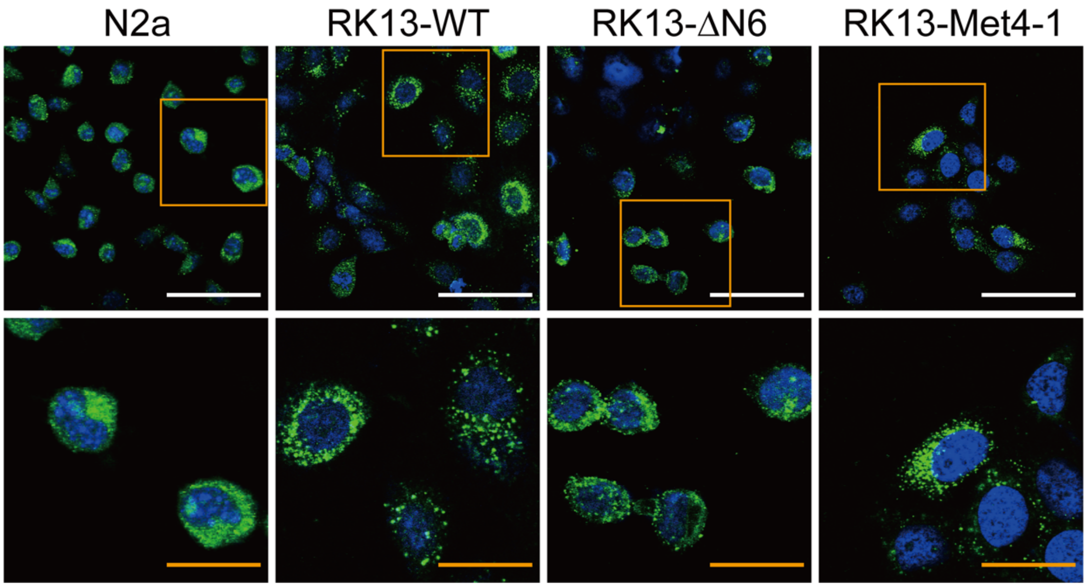

Figure S1. Immunofluorescence staining of RK13 cells stably expressing various PrP forms. Selected areas (yellow boxes in upper panel) of merged images of DAPI (blue) and PrP staining (green) in N2a, RK13-WT, RK13-Met4-1, and RK13-ΔN6 cells in figure 3A were enlarged (lower panel) to show the localization of PrP. All cells were stained with 3F10 anti-PrP antibody. White scale bars = 100 μm; yellow scale bars = 40 μm.

Figure S2

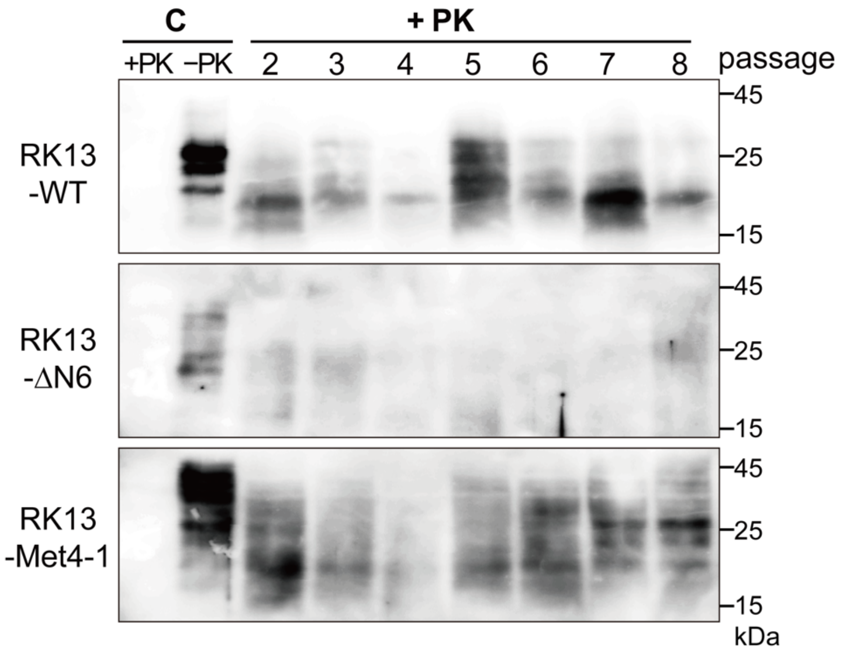

Figure S2. The original image of figure 4B. In all images, the PK digested (PK+) control (C) cell lysates were loaded in the same lane of molecular-weight marker. Handwritten numbers of molecular weight erroneously appeared in these lanes of Western blot images.

Figure S3

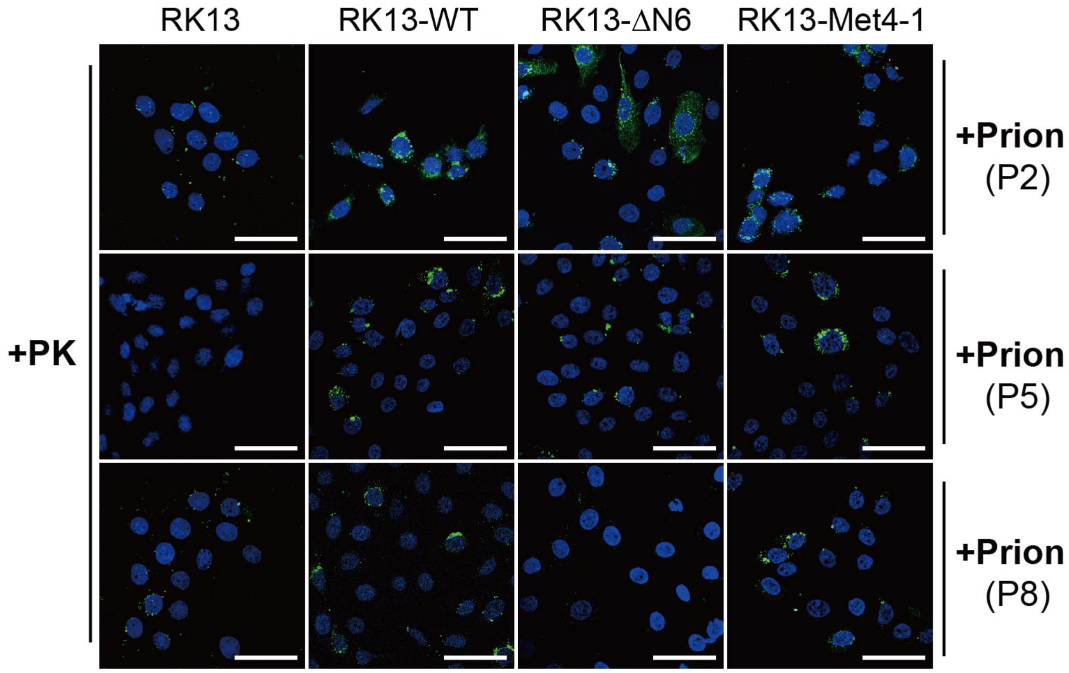

Figure S3. Immunofluorescence staining of PK-resistant PrP in prion-infected RK13, RK13-WT, RK13-Met4-1, and RK13-ΔN6 cells. Passages 2, 5, and 8 (P2, P5, and P8) cells were digested with PK and stained with 3F10 anti-PrP antibody (green). Nuclei were stained with DAPI (blue). The images of P5 cells, which were shown in figure 4E, were included here to show changes over passages. Scale bars = 100 μm.

Figure S4

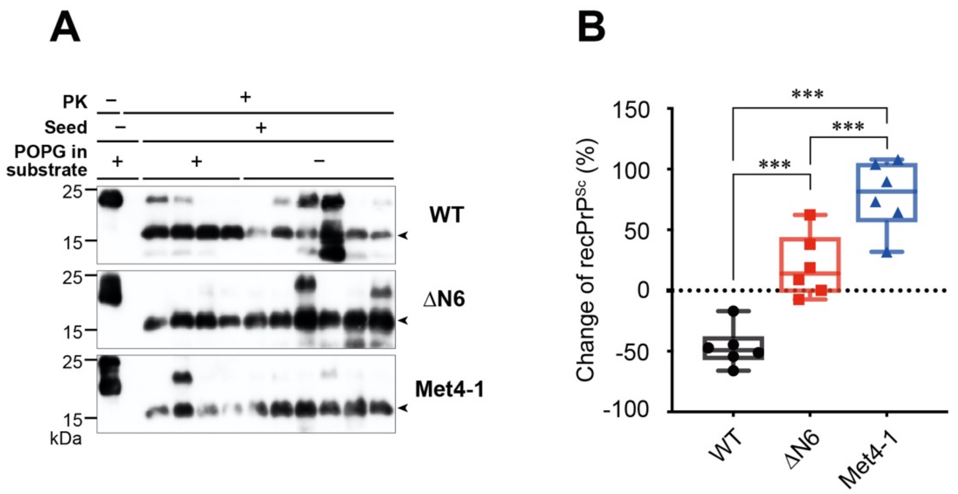

Figure S4. Generation of WT, Met4-1, and ΔN6 recPrP^{Sc} by 1 round PMCA with regular or reduced POPG. (A) WT, Met4-1, and ΔN6 were subjected to one round PMCA with or without POPG in the substrate as indicated. The PK-resistant PrP bands were indicated by arrowheads. Each lane represented an individual PMCA reaction. PrP was detected by Western blotting with the 3F10 anti-PrP antibody. **(B)** Quantification of the changes of PK-resistant WT, Met4-1, or ΔN6 PrPs generated by PMCA with or without POPG in the substrate (n = 6). Statistical significance was determined by one-way ANOVA followed by Tukey's multiple comparison test. *** represents $P < 0.01$. Error bars indicate standard deviations. Each point in B represents a single PMCA reaction.

Supporting text for figure S4: To determine whether altered POPG-binding capabilities of NPR mutants influence recPrP conversion in PMCA, we compared their propagation in one round PMCA with regular or reduced POPG in the substrate. In reactions with reduced POPG, the final POPG concentration was 10% of that in regular PMCA, which is because

1) there was no POPG in the substrate (90 μl) and 2) the seed, which was 10 μl of previously generated PMCA product, contained regular amount of POPG. All seeded reactions with regular or reduced POPG were able to convert recPrP to PK-resistant PrP (Supporting Fig. S4). Densitometric quantification of the signature PK-resistant band (pointed by arrows) showed that much less amount of WT (~47%) was converted to PK-resistant form in reactions with reduced POPG (Supporting Fig. S4; Supporting table S1). In contrast, with reduced POPG, Met4-1 and ΔN6 had ~79% and ~20% increase in PK-resistant PrP, respectively. It is important to note that the PK-resistant PrP detected here was not necessarily the infectious PrP form (38,39), but they were clearly converted from PK-sensitive to PK-resistant conformation during the PMCA and importantly, the appearance of a similar sized PK-resistant fragment (15 kDa) suggested their conformations were similar to that of recPrP^{Sc}. If the NPR were not important for its binding to POPG or the NPR-mediated POPG-interaction were not important for the conformational change during PMCA, the NPR mutants would have behaved in a similar manner as WT in PMCA reactions with reduced POPG, but clearly, this was not the case. Thus, the opposite effects of reduced POPG on WT and NPR mutants suggested that the altered POPG-binding capability of NPR mutants affects their conversion in PMCA. It is also consistent with the idea that NPR is important in PrP-POPG interaction.

Supporting tables

Table S1. Conversion of recPrP with regular or reduced POPG.

Average PK-resistant band intensity			$\frac{(-\text{POPG}) - (+\text{POPG})}{(+\text{POPG})}$
Group	+POPG	- POPG	Ratio (%)
**WT**	83.79	44.64	-46.72
**ΔN6**	45.28	54.42	20.19
**Met4-1**	63.94	114.21	78.62

Table S2. Scores of spongiosis (vacuoles/mm^2).

**Vacuoles-Score Scale**	
**Vacuoles/mm^2**	**Score 0−5**
X>725	5
650<X≤725	4.5
575<X≤650	4
500<X≤575	3.5
425<X≤500	3
350<X≤425	2.5
275<X≤350	2
200<X≤275	1.5
125<X≤200	1
50<X≤125	0.5
X≤50	0
